# Enhanced coupling of perovskites with semiconductive properties by tuning multi-modal optically active nanostructured set-ups for photonics, photovoltaics and energy applications

**DOI:** 10.1039/d5ra00458f

**Published:** 2025-02-25

**Authors:** Shaimaa Elyamny, A. Guillermo Bracamonte

**Affiliations:** a Departamento Académico, Facultad de Ciencias Químicas, Universidad Nacional de Córdoba X5000HUA Córdoba Argentina guillermobrac@yahoo.ca gbracamonte@unc.edu.ar; b Consejo Nacional de Investigaciones Científicas y Técnicas (CONICET, Instituto de Investigaciones en Fisicoquímica de Córdoba (INFIQC) X5000HUA Córdoba Argentina; c Electronic Materials Research Department, Advanced Technology and New Materials Research Institute, City of Scientific Research and Technological Applications (SRTA-City) New Borg El-Arab City, P.O. Box 21934 Alexandria Egypt

## Abstract

This review describes the coupling of semiconducting materials with perovskites as main optically active elements for enhancing the performance depending on the optical set-up and coupling phenomena. The various uses of semiconductor nanoparticles and related nanomaterials for energy conduction and harvesting are discussed. Thus, it was obtained different materials highlighting the properties of perovskites incorporated within heterojunctions and hybrid nanomaterials where varied materials and sources were joined. Different multi-layered substrates are reported, and different strategies for improved electron and energy transfer and harvesting are elucidated Further, enhanced coupling of semiconductive properties for the above-mentioned processes is discussed. In this regard, various nanomaterials and their properties for improving energy applications such as solar cells are demonstrated. Moreover, the incorporation of plasmonic properties from different noble metal sources and pseudo-electromagnetic properties from graphene and carbon allotropes is discussed. Since variations in electromagnetic fields affect the semiconductive properties, it leads to varying effects and potential applications within the energy research field. Hence, this review could guide the development within energy research fields as nanophotonics, photovoltaics, and energy. This review is mainly focused on the development of solar energy cells by incorporating perovskites with varied hybrid nanomaterials, photonic materials, and metamaterials.

## Abstract 2

This review describes the coupling of semiconducting materials with perovskites as main optically active elements for enhancing the performance depending on the optical set-up and coupling phenomena.

## Graphical abstract

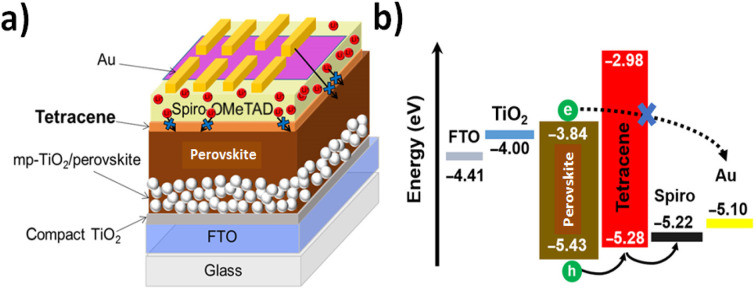

## 1. Introduction to conductive, semiconductive, and energy harvesting nanomaterials

Semiconductive properties can be derived from different types of materials on different scales. In this regard, different phenomena such as conduction,^1^ harvesting,^2^ signal translation and transductions,^3^ and encryption^4^ of different modes of energy^5^ can be developed. Thus, thermal,^6^ electronic,^7^ photonic,^8^ quantum,^9^ and luminescence properties^10^ can be developed and controlled for varied applications. It should be mentioned that semiconductor materials can exhibit dual properties of conduction, similar to copper, and insulation, similar to glass, based on their intrinsic electronic constitutions and controlled conditions of study.^11^ Therefore, conductor- and semiconductor-nanomaterials based on their particular electronic band gaps allow the mobility of particular energy modes. These band gaps related to electronic energy levels could be tuned, controlled, modulated, and influenced in different ways depending on the nanomaterial under study.^12^ Moreover, it is known that in some materials, these band gaps could be generated by opto-stimulation, and by this manner, opto-electronics and energy applications were developed. Therefore, various classical and non-classical light sources could be applied to generate varied mobility of energy modes through the material.^13^ The energy modes could be varied and produced from different physical phenomena; for example, high-intensity electromagnetic fields could be produced from metallic and organic materials, where high-density electronic waves modify their surroundings.^14^ In a similar manner, enhanced light scattering phenomena produced from porous and nano-holed materials^15^ could show drastic effects for the development of laser properties.^16^ In this regard, well-known semiconductive materials as well as those produced by the combinations of them for achieving coupling phenomena and enhanced effects should be mentioned. Different materials involved in current developments towards the next generation of semiconductive materials such as; (i) organic; (ii) inorganic; (iii) hybrid; and (iv) metamaterials are highlighted. Among these material groups, (i) highly conjugated carbon-based chemical structures, such as graphene,^17^ carbon nanotubes,^18^ derivatives and carbon allotropes;^19^ (ii) varied atomic inorganic constitutions of quantum dots based on non-metallic atoms, such as Zn, Cd, Te, and Se,^20,21^ as well as carbon-based quantum dots;^22,23^ metallic and non-metallic nanoparticles,^24,25^ plasmonic materials,^26^ and photonic materials with variable constitution with the incorporation of varied groups of elements from the periodic table;^27^ and (iv) metamaterials^28^ are worth mentioning.

From these different sources of semiconducting materials, perovskites are promising for energy and solar cells^29^ and related applications.^30^ In the past few years, the efficiency of perovskite solar cells increased drastically, from around 3% in 2009 to over 25% in 2022.^31,32^ It is known that the perovskites such as ABO_3_ (MgSiO_3_, FeSiO_3_ and Al_2_O_3_) ranging from natural sources such as silicate perovskites (MgSiO_3_)^33^ to synthetic perovskites with variable atomic constitutions showed difference in the chemical structure and crystalline packaging.^34^ This variation in chemical conformations and geometries is favourable for the inclusion of variable additional chemical species, atomic defects, ions, *etc.*, which affected the structures and properties. In particular, controllable and accurate perovskite chemical structures showed lower energy voltages of band gaps that opened up avenues for its study and use in energy applications. For example, methylammonium halides, among which the most common halide is methyl ammonium triiodide (CH_3_NH_3_PbI_3_), showed interesting properties for dye-sensitized solar cells for the generation of free electrons and holes, with consequently improved electronic conductions, and diminution of energy losses.^35^ In this way there is huge interest in this direction to tune their band gaps towards optimal energy voltages to be used for solar cell applications.^36,37^ Thus, other atomic constitutions and strategies were assayed to tune their Optoelectronic properties. Inorganic halide perovskites of different atomic constitutions were joined to reduce the band gaps by proposing donor–acceptor pair codoping such as Sn- and Pb-based structures (CsSnBr_3_ and CsPbI_3_). This combination allowed band gaps of 1.2 and 1.1 eV that are so close to optimal values for solar cells.^38^ In this manner, the donor–acceptor strategy showed augmented solar energy absorptions in comparison to individual components. In a similar manner, other strategies could affect the energy band gaps with potential material properties for energy applications. However, the development of new approaches and strategies applied with these types of nanomaterials in order to improve the properties for targeted uses is still of interest. In addition, different designs, modifications of substrates, and approaches are discussed along this review. Consequently, different strategies are developed for improved electronic properties of energy devices, nanophotonics, photovoltaics, and solar cells.^39^ In this regard, it was afforded to combination of different types of different types of semiconductors highlighting some ones very well-known such as graphene and related semiconductor materials. For example, directly integrated mixed-dimensional van der Waals graphene/perovskite heterojunctions are notable for fast photodetections.^40^ Further studies achieved improved performances depending on targeted uses.

The coupling phenomena^41,42^ involve enhancing electron conduction and harvesting. This enhanced coupling was endowed by the incorporation of various nanomaterials and properties. Thus, high-electromagnetic fields within the near field showed potential uses in the solar cell technology, such as plasmonic materials. Moreover, other sources of electromagnetic fields such as graphene^43^ and modified carbon-based chemical structures as carbon nanotubes^44^ are showed and discussed herein. In addition, quantum coupled properties^45^ and electron shuttles^46^ were contemplated. Therefore, hybrid composites were endowed with these properties by the incorporation of various semiconductive nanomaterials to tune new properties and applications. So, it was afforded to improved solar cells approaches such as perovskites solar cells; as well it was showed studies towards optoelectronics,^47^ nanoelectronics,^48,49^ and nano-, micro-circuits.^50^

Although perovskite solar cells showed competitive power conversion efficiencies (PCE) and the potential for improved performance, they are less stable than the leading photovoltaic (PV) technologies. When perovskites react with moisture and oxygen or are exposed for a lengthy period of time to light, heat, or electrical current, they can degrade. Researchers are investigating degradation in both the perovskite material itself and the surrounding device layers to boost stability. In order to create commercial perovskite solar products, cell endurance must be improved. Creating uniform testing and validation methodologies is a challenge when evaluating degradation in perovskites. Various encapsulation techniques, atmospheric compositions, illumination, electrical bias, and other parameters are tested under a wide range of conditions, and research groups provide performance results based on these results.^51^

## 2. Perovskite semiconductive properties

Perovskites due to their intrinsic chemical structures showed a large variety of physical and chemical properties with impact in the material science oriented toward opto-and electro-responsive material developments. Thus, the ABO_3_ perovskite chemical structures have a cubic structure where B as the cation such as Sn^2+^, Pb^2+^, Ge^2+^, Bi^3+^ has in six fold coordination by eight anions forming an octahedron; in addition to A is an organic or inorganic cation, FA^+^, MA^+^, Cs in 12 fold cuboctahedral coordination and X is a halide anion as Cl^−^, Br^−^, or I^−^.^52^ This 3D atomic spatial disposition gives rise to variable stabilities depending on the atomic composition, accompanied by different properties and potential uses. Moreover, this structure could be doped with other atoms and chemical species within interstices and inter-layers to modify their properties.^53^ The power conversion efficiency (PCE) of perovskite solar cells based on leads increased from 3.8% in 2009 to 25.7% in 2022 as a result of their excellent photovoltaic properties, which include an adjustable band gap, high carrier transport mobilities, long hole–electron diffusion lengths, and small exciton binding energies.^54^ However, in 2024, triple-junction perovskite–perovskite–silicon solar cells with a power conversion efficiency of 24.4% in 2024 was developed;^55^ and the highest PCE for perovskite solar cells has reached 26.7% recently in 2025.^56^ Thus, the multi-layered architectures and bottom up showed drastic differences in the performances achieved. Accordingly, the accurately deposited materials by controlling the concentrations and interphases within the molecular and nanoscale level showed differences in the developed opto-electronics.

In this manner, perovskites, depending on their atomic compositions and media, could generate: (i) photoluminescence; (ii) electron conductions; (iii) photonics transmissions; (iv) photo-electron conductions;^57^ (v) electromagnetic fields nominated as plasmons; and (vi) other phenomena by their accurate incorporation within shorter distances in the nano-scale with varied nanomaterials with variable properties. Therefore, there are a large number of properties to be exploited, studied and applied in the design of new photo-active nanomaterials and metamaterials.

It could be mentioned that for light emitting diodes (LEDs), organic–inorganic hybrid halide perovskites were applied by varying the molecular bridging ligands.^58^ Therefore, the molecule dissociation energy of the Dion–Jacobson structure by the incorporation of the 1,4-bis(aminomethyl)benzene molecules was two times higher than that of the typical Ruddlesden–Popper (RP) structure based on phenylethylammonium ligands. Thus, these LEDS based on these improved dissociation energies showed half-life time over 100 hours, which was at least two orders of magnitude longer than the referenced Ruddlesden–Popper structural quasi-two-dimensional perovskite.

In the incorporation of semiconductors, it is important to highlight the design and 3D architectures of the materials. For example, the epitaxial growth is a technique used to tune perovskite substrates, which can generate high-quality crystals with properties for desirable and continuous optical activity.^59^ Thus, the crystallinity and homogeneous patterns produce constant properties from the atomic, molecular and higher sized substrates. While the addition of atomic defects could be desirable in many cases, it should be fully controlled for targeted new properties. Perovskite materials are clear examples of these highlights.^60^ In addition, as it is known the effect of shapes and geometries of nanomaterials showed important effects in electronics and quantum properties. For example, semiconducting nanowires presented potential applications within optoelectronic and photonic applications. The nanowires and nanotubes have anisotropic properties based on the different electronic oscillations along their nano-surfaces. Therefore, for example, inorganic nanomaterials such as CdS quantum dots that are optically pumped showed nanolaser properties.^61^ In a similar manner, perovskite nanowires showed particular anisotropic properties.^62^ Thus, vacancies of halogen afford the ligand-assisted self-assembly of perovskite quantum dots within nanowires,^63^ tuning, by this manner, the optoelectronic properties. In this research work, the design of 3D-printed nanocomposite inks produced high-luminescence emitting colloidal cesium halide perovskite (CsPbX_3_, X = Cl, Br, and I) nanowires embedded in a polymeric matrix. Thus, the nanowire geometry formation was a programmed print path that allowed highly polarized absorption and emission from modified polymeric films. In this context, it should be noted that perovskites and semiconductors, in general, never acted alone, and at the place always in combination with other optically active materials.^64^ This type of approach is related to optically active metamaterials with different applications for opto-electronics, micro-, and nano-circuits^65^ with low-energy applications required^66^ for stimulation. Moreover, the optical lens could be tuned for imaging, holography and optical detections.^67^ Many developments in progress show broad interest in different research fields where in all the cases it should be contemplated tuning of semiconductive properties, evaluate stabilities; improve signaling caused energy dissipation or losses. Therefore, all the mentioned variables were of importance and they were considered for photo-active materials used in energy applications such as solar cells as well. In addition, in particular, it is known that perovskites showed high-impact applications within this current research field due to their particular properties associated with smaller differences of potential energy bandgaps required for optical stimulation. In this way, as it is known for optimal efficiencies of perovskite solar cells (PSCs), energy losses produced by different mechanisms such as interfacial defects should be minimized. In this context, controlling architectural constraints for optimal interfacial contacts could lead to band alignments at the buried interfaces of perovskites, where it is crucial to minimize energy losses.^68^ Therefore, achieving optimal interfacial contact and band alignment at the buried interface of perovskites is crucial for minimizing the energy loss in perovskite solar cells.

**Fig. 1:**
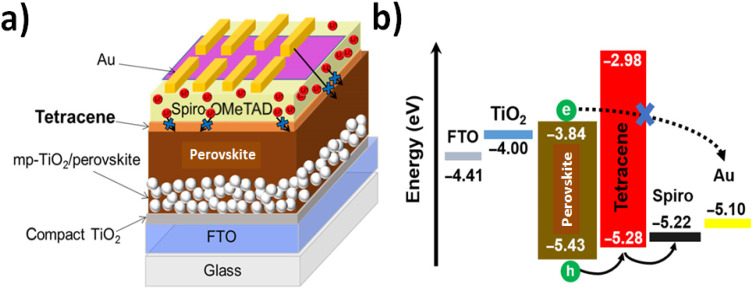
Solar cell architecture and energy diagram. (a) Schematic of the device architecture and (b) energy-level diagram obtained from UPS measurements of a complete solar cell with graded doped HTLs of undoped tetracene with a doped spiro layer. In the schematic, the red spheres are representative of Li^+^ ionic dopants present in a doped spiro layer, and the short and long arrows demonstrate the extrinsic ionic and Au migrations that are blocked by the undoped tetracene layer. The perovskite composition is Rb-passivated Cs_0.06_FA_0.79_MA_0.15_Pb(I_0.85_Br_0.15_)_3_. Reprinted with permission from ref. 66 (Q. Zeng *et al.*). Copyright 2019 Scientific Reports, Springer Nature.

For significant improvements, a rubidium-doped perovskite with a triple-cation coordination [*e.g.*, Rb-doped Cs_0.06_FA_0.79_MA_0.15_Pb(I_0.85_Br_0.15_)_3_] incorporated in a multilayered device with varied nanomaterials was designed.^69^ First, an evaporated tetracene free of dopant agents (120 nm) on top of the perovskite layer, capped with a lithium-doped spiro-OMeTAD layer (200 nm) and a top gold electrode, allowed minimal interfacial defects. Thus, the photoluminescence of the perovskite layer interfaced between a graded hole transport layer (HTL) and a mesoporous TiO_2_ electron-extracting layer reached 15% in comparison to 5% for the perovskite layer acting as the interface between TiO_2_ and a spiro-OMeTAD alone. Therefore, an efficiency up to 21.6% accompanied by an extension of the power output of over 550 hours of continuous illumination (Fig. 1) was demonstrated.

As it could be observed in the intrinsic design and composition, the materials incorporated showed electron transferences between different levels of energies and bandgaps. These processes could be affected by other materials incorporated in the close surrounding such as electromagnetic and pseudo-electromagnetic fields.^70^ Thus, electron shuttle could be achieved with modified performances that should be tested. In these perspectives, there are many modifications that could be explored looking for enhancements in the middle of the whole energy translation through space and time.

In a similar manner, it should be mentioned that the importance of the optical design approach is related to the nanomaterials incorporated within a substrate. Moreover, the important role of the 3D structure in the photo-conversion and stimulated and targeted phenomena was demonstrated. Therefore, unless there is one event of electron recycle from the electron–hole pair towards photon to electron–hole pair by solar light excitation, these phenomena could show different efficiencies depending on the substrate bottom up strategies. For example, it was determined that for an estimated internal efficiency of 70%, the experimentally values were measured around 15% in planar films; while the value raised to 57% when it was generated from textured substrates.^71^ This fact was explained by the internal recombination processes, and photo-excited carrier dynamics. Thus, it was concluded that the modified layers had the ability to improve power conversion efficiencies for LEDs and solar cells.

In order to understand the photophysics of hybrid materials and final efficiencies controlling the incorporation of diverse semiconductors, the physics behind lower photon conversion phenomena should be understood. For example, extensive characterization of optoelectronic devices tuning optoelectronics was carried out. Moreover, key measurement was carried out to evaluate charge extraction in the presence of different semiconductive material joints. Thus, an optoelectronic device coupled with an oscilloscope was used to measure the duration for the charge to rise and then flow out of the device for optimizing and tuning the efficiency. It was observed that in the perovskite-sensitized devices, the current decay was much faster than that in the dye-sensitized devices, suggesting faster charge extraction with the perovskite incorporations. This made the conclusion about the role of perovskites in relation to a dual function focused on efficient light absorbing properties and facilitating electron transport as well.^72^ Therefore, nanomaterials, optical properties, and architectures should be accurately controlled to study the opto-electronic properties for tuning non-classical light and electronics pathways. The tuning involves many cases by coupling directional opto-electronic flows. For example, suitable absorbing materials have recently been designed for tuning the photodetection in the near- and mid-infrared spectrum. Thus, an asymmetric resonant waveguide was fabricated with the incorporation of graphene that led to evolution of new properties related to a perfect metamaterial absorber. In this way, it was induced photo thermoelectric directional photocurrents within asymmetrical channels.^73^ In this approach it should be highlighted that the extraordinary optoelectronic properties achieved with zero-band gaps showed the capability to absorb, transfer, and conduct through the metamaterial for the photodetection event in the infrared spectrum.

Improving the optical and semiconductive properties by controlling other variables gained interest. The incorporation of electromagnetic fields of high intensity to produce varied electronic phenomena with potential perspectives of enhancing properties could be highlighted. In this direction appeared hybrid materials, metamaterials, and plasmonics materials incorporated within new types of materials.^74^ In particular, the proof of concept and approaches based on the combinations of nanomaterials and properties by varied chemical methodologies as well as other technologies are discussed. Therefore, how coupling photonics, electronics, band gaps, electromagnetic fields, and other phenomena by varied manners can tune, improve and enhance emissions, conductions and efficiencies is demonstrated.

## 3. Semiconductive properties within heterojunctions

Heterojunction formation and directional flow of energy are excellent optical approaches from Optical and chemistry benches for the development of technology.^75^ Therefore, they were in the beginning related to a concept of joining different nanomaterials with similar energy bandgaps affording electron shuttling with improved efficiencies in photovoltaics and energy applications.^76^ However, this new nanomaterial design and fabrication is currently used to join different types of materials with variable optical properties. Therefore, in many cases along the discussion of results it was not mentioned the approach used due to the importance of phenomena involved and studied. In this perspective, this section intended to briefly discuss these topics highlighting the semiconductive heterojunction-based nano-composites and substrates. However, the accurate control of their fabrication is still a challenge. In this context, different methodologies could be reported for heterojunction design by deposition of varied film compositions such as molecular beam epitaxy^77^ or chemical vapor deposition (CVD).^78^ In a similar manner for multi-layered depositions was proposed molecular stacking by van der Waals interactions.^79^ Graphene, a highly conjugated chemical structure, was proposed to be used by this methodology due to its high electronic density from π-orbitals that allowed non-covalent interactions nominated as π–π stacking. Then, the combination of graphene and perovskites showed enhanced performances based on their intrinsic properties incorporated within multi-layered bottom ups such as heterojunctions. Recently, it has been reported that 2D perovskite–graphene layered composites can be tuned for photocatalysis.^80^ In this investigation, the formation of assembled ultrathin exfoliated Dion–Jacobson perovskite layers with reduced graphene oxide layers was demonstrated for the first time. By this approach, a high nanostructured layered surface of nanocomposite based on tailored electrostatic approaches was obtained. This fact showed the importance of the intrinsic material's optical properties as well as chemical surfaces to lead non-covalent interactions affording spontaneous depositions. Moreover, the electron properties of the layered perovskite–reduced graphene oxide composites were evaluated with the incorporation of various lanthanides as A-site cations in the Dion–Jacobson perovskites, including LaNb_2_O_7_ (LNO), PrNb_2_O_7_ (PNO), and NdNb_2_O_7_ (NNO). Applying this multi-variable controlled approach, higher performances in photocatalytic H_2_ production was demonstrated, achieving a hydrogen evolution rate of 835 μmol g^−1^ under light illumination. The clear improved performances in the presence of all the mentioned components in comparison to control experiments in the absence of at least one of them were attributed to the optimal interfacial effects that diminished opto-electronic losses.

As it was shown in many reports, the design and bottom up of the semiconductor incorporation in the optically active composite showed drastic differences in their performances. Therefore, the chemistry and methods associated are very important. Thus, it is a factor to evaluate up on needs depending of the targeted study or application. Thus, by wet-chemical methods, semiconductor nanomaterial-based epitaxial heterostructures were obtained.^81^ Thus, accurately nano-sized particles with control of shapes and 3D geometries with the incorporation of well-differentiated varied materials in the structure obtained were reported.

After developed all these different methodologies, it was not stopped the research and developments from the control of the nanoscale towards higher sized dimensions. This fact could be explained by the potential of heterojunctions by modifying their structures and material assemblies. In this perspective, many semiconductive materials were used to fabricate heterojunctions for energy applications. For example, silicon heterojunctions by the incorporation of multi-layered silicon oxides within wafer-type materials should be highlighted.^82^ In order to understand the capability of this technology, it could be noted as for example the resolution in the design related in P-type and n-type silicon wafers, with thicknesses of 300 μm, were covered by a 60 nm thick silicon nitride (Si_3_N_4_) layer deposited by plasma-enhanced chemical-vapor deposition (PECVD). Moreover, the incorporation of Ti (20 nm)/Pt (100 nm) layers on the back of the electrodes, which in contact with semiconductive layers defined ohmic junctions. In addition, on the top of Si_3_N_4_, two circular Pt/Ti electrodes with a diameter of 1 mm at 4 mm away from each other were deposited. Therefore, an idea about how important are sizes, lengths and materials inter-connected and how it could be potentially tuned with the incorporation of new optically active nanoplatforms could be achieved. In this regard, as it is known, silica chemical structures showed excellent dielectric and optical properties, as well as semiconductive properties to be tuned. Moreover, the use of these materials as support as well as platform to be modified for further developments was due to their relative easy malleability, methodologically manipulated, and synthetic pathways developed.^83^ Therefore, silicon heterojunctions were developed for photodetectors and solar cell applications. In brief, this approach was based on the multi-layered deposition of silicon oxide substrates known as wafers with variable chemical composition contained between two electrodes.

**Fig. 2:**
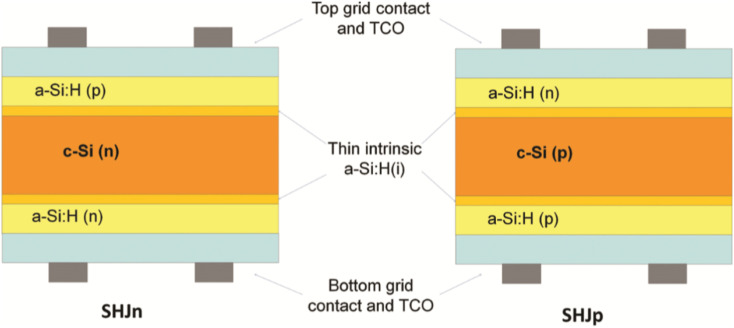
Silicon heterojunction solar cells on n-type silicon (SHJn) and n-type silicon (SHJp) hetero-junctions with intrinsic thin-layer (HIT) solar cells. Reprinted with permission from ref. 84 (M. Mikolášek *et al.*). Copyright 2017 Book: Nanostructured Solar Cells, Chapter 4, Intech CC.

Thus, varying material composition could be achieved by varying the efficiencies. Thus, modifications of silica substrates as amorphous silicon carbides as well as other modified silicon substrates to tune absorption and emission band-gaps were applied. Moreover, modifying the material compositions of electrodes at the bottom of the cell could reduce energy losses. In addition, these modifications allow replacing electrode materials associated with higher costs, such as Indium Tin Oxide (ITO), for those cheaper as Zinc Oxide (ZnO) and Indium-Zinc Oxides (IZO) (Fig. 2).^84^

It should be noted the expansion of the designs using silicon based modified substrates due to their excellent optical properties. It afforded to join varied materials by different laser-assisted techniques,^85^ electrochemical deposition, as well as wet chemistry.^86^ Therefore, this bottom-up strategy afforded highly sensitive optical set-ups by just controlling the passivation of surfaces and improved packaging that generated a gain conversion with an absolute efficiency of up to 0.3%. This improvement was explained by the reduced density of recombination-active interface states and demonstrated by a time-dependence phenomenon associated with a developed kinetic model.^87^ In the same way, the importance of the crystalline silicon oxide growth by plasma processes to (i) eliminate the pattern of one of the doped layers with collective layers, and (ii) improve electron–hole contacts by the passivation of surfaces was demonstrated. These facts led to reduced energy losses through contacts and surfaces. Thus, a proof-of-concept 9 cm^2^ tunnel-interdigitated back-contact solar cell with a conversion efficiency higher than 22.5% was obtained.^88^ This reported value was considerably higher than that of previous ones, for example, with 12% with amorphous Si/polycrystalline Si stacked solar cells.^89^ However, the main variables for silicon heterojunction solar cells with high performances were discussed and are of interest for new designs, bottom-ups and proof of concept.^90^ After the award assignation to this approach by the Nobel Prize in Physics 2000, shared by USA and Russia, for “developing semiconductor heterostructures used in high-speed-photography and opto-electronics”,^91^ It still being a challenge to improve it by joining varied nanomaterials and 3D accurate control of geometries, and assemblies.

**Fig. 3:**
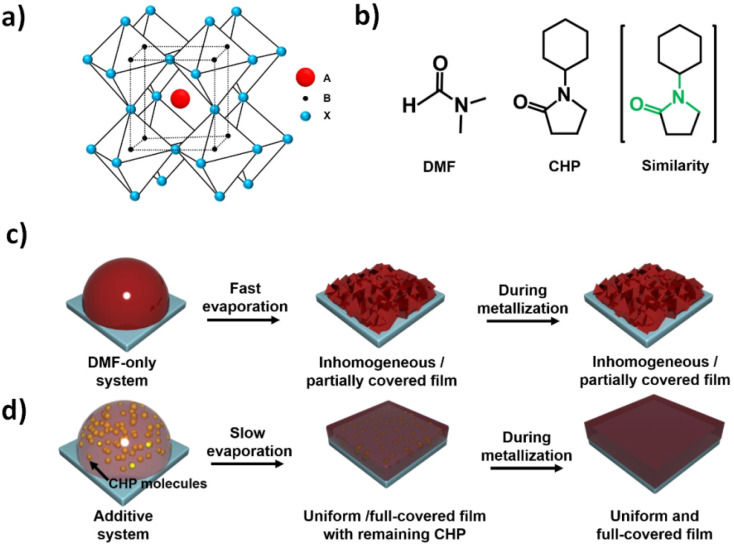
Schematic of the perovskite film formation: (a) illustration of the crystal structure of CH_3_NH_3_PbI_3_ perovskites and (b) chemical structures of DMF and CHP. Schematic of perovskite film formation in (c) DMF only and in (d) a CHP additive system. Reprinted with permission from ref. 96 (R. Kang, *et al.*). Copyright 2014 Sci. Rep. Springer Nature.

In order to incorporate more materials participating in many requiered aspects, it should be mentioned the incorporation of ionic liquids, either as additives to perovskite precursor solutions or as alternatives to traditional precursor solvents.^92^ The power conversion efficiencies (PCEs) have already reached around 25% in this way; however, to the best of our knowledge, the long-term stability issue of such devices still impedes their commercialization. Moreover, with these perspectives, other designs of varied hetero-structures by joining varied semiconductive nanomaterials should be mentioned. For example, the introduction of electrons by the incorporation and excitation of CdSe quantum dots into TiO_2_ nanoparticles,^93^ and the use of tuned nanostructures such as CdS/ZnSe core–shell nanoarchitectures.^94^ In a similar manner, the incorporation of other semiconductors such as graphenes for the fabrication of heterojunctions was reported to shed light on emerging photovoltaics.^95^ Thus, their electronic and spectroscopic properties were exploited for tuning opto-electronics within heterojunctions. In this manner, it was showed how these approaches could be used to study and evaluate properties from the nanoscale towards higher sized substrates for solar cell energy applications. Therefore, it led to the accurate control in the design, synthesis and fabrication of varied heterostructures based on the materials as well as the conditions and methodologies applied. As for example, about conditions and incorporated material, it was used small quantities of *N*-cyclohexyl-2-pyrrolidone (CHP) within *N*,*N* dimethylformamide (DMF) as a morphology controller of the homogeneous film deposition on planar perovskite solar cells (PSCs) substrates. This technique allowed controlling the previous inhomogeneity observed by other techniques in comparison to vacuum deposition methods. However, the use of a more accessible and low-cost method by wet chemistry (Fig. 3) should be highlighted in order to solve one of the highest challenges of planar heterojunction (PHJ) perovskite photovoltaic technology.^96^

In addition, planar-heterojunction perovskite solar cells based on a two-step deposition process with high performances were reported.^97^ By this methodology, the deposition of CH_3_NH_3_PbI_3_ films that afforded a reproducible conversion efficiency of 13.53% of photoelectric events was improved. The higher efficiency achieved by this optical approach showed the importance of the high quality of the multi-layers added in the photonic device by increasing the efficiencies of individual functions of incorporated layers. This approach later was even improved and still being under focus. Recently, MAPbI_3_-based perovskite solar cells achieved an efficiency exceeding 21% *via* aging treatments by wet chemical methods.^98^

Thus, theorical calculations and experimental results demonstrated the behaviour mechanism by studying the optical coupling, transport of carriers, and thermodynamic characteristics. And by this manner, it was discovered the origin of energy losses. Therefore, six categories of microscopic heat conversions were recorded, where the Joule and Peltier heats were defined as intrinsic losses in the heterojunctions perovskite solar cells (PSCs). Thus, the manipulation methods to diminish the energy losses are discussed. Therefore, controlling manipulations and managing concentrations for the doping and energy level alignments achieved enhancements from 21.37% to 23.84%.^99^ In the same line, thermal modeling of perovskite solar cells by tuning electron and hole transfer layers has been recently reported, highlighting enhancements up on 24% but considering the heat generated due to losses resulting from resistive heating and non-radiative recombination. The thermal behavior of devices with controlled structures with geometrical dimensions of a practical PSC deposited on a glass substrate with silane passivation layers showed operating temperatures until 84.5 °C depending on the applied electron transfer material. Thus, solar cells are versatile considering long-term and heat-related losses.^100^

In addition it is highlighted the interest of the incorporation of plasmonics metal-semiconductors heterojunctions towards maximum efficiencies for solar energy conversions.^101^ This study allowed by using the model of density matrix the relation between the scattering, hot electrons, and dipole–dipole coupling through plasmonic dephasing, incorporating the nominated coherent and incoherent dynamics required for plasmonic interactions in the time scale. This model was extended to the calculation of the Shockley–Queisser limit for conversions on photovoltaics and solar-to-chemical processes. By this manner, the application of each plasmonic enhancement produced by the energy of the plasmon, semiconductor, and plasmon dephasing was allowed. These theoretical calculations could be extended for variable semiconductor materials added within heterojunctions.

In the last years, it was increased the interest towards developments with the incorporation of plasmonics nanostructures such as plasmonic-enhanced light harvesting and perovskite solar cell performance using Au biometric dimers with broadband structural darkness^102^ hybrid perovskites have recently attracted enormous attention for photovoltaic applications, and various strategies related to light management and photo carrier collection are developed to enhance their performance. As an effective route toward near-field light enhancement, metal nanostructures with subwavelength dimensions can couple incident photons with conduction electrons, giving rise to localized surface plasmon resonances. They have been even recently evaluated by incorporating biocompatible gold nanoparticles for wearable optoelectronic applications. Thus, it was showed how sensitive could be these phenomena tuned.^103^

In this manner, varied joined plasmonic materials, as well as new modes of high electromagnetic fields sources were reported within this type of bottom up. For example, the combination of mesoscopic graphene/perovskite heterojunctions,^104^ and TiO_2_/noble metal nano-heterojunctions were reported.^105^ Thus, graphenes show particular properties: (i) acceptable electromagnetic field intensities^106^ and enhanced fields too,^107^ (ii) special electronic conductions comparable to copper,^108^ and (iii) quantum properties; afforded to tune perovskite heterojunctions.^104^ This heterostructure showed high UV absorption generated from perovskite hot electron transport *via* hole transport through porous graphene layers. Thus, it should be stated clearly that the introduction of graphene as a source of multi-functional nanomaterial with the capability to participate in opto-active phenomena by different pathways highlighted the generation of pseudo-electromagnetic fields. Therefore, graphene acted as the collecting material and channel of carriers for electron transport through its electronic waves. This charge transfer process was controlled by luminescence measurements. This material characterization afforded hybrid films for phototransistor fabrication based on a graphene/perovskite heterojunction. This device performed with a high sensitivity and specificity of 2.0 × 10^3^ A W^−1^ and 7.2 × 10^10^ Jones, respectively. Moreover, based on the incorporation of TiO_2_/noble metal nano-heterojunctions,^105^ increased hot electron production was observed from their nanostructures due to the incorporation of photo-active TiO_2_ surrounded by electromagnetic fields from metallic nanoparticles. So, it was highlighted its application for photocatalysis as well as solar energy. It should also be noted that all these properties are produced by the proper excitation of the material to generate nano-optics and specific signalling produced from confined nanostructures and surfaces.

In this perspective, the heterojunctions as tuneable heterostructures led to theoretical and experimental results for energy applications from the nanoscale, to the micro-scale and higher sized substrates. It was highlighted the optimization of coupled properties such as varied energy modes within the quantum, and nanoscale, with different semiconductors. In this way, in the next section, the coupling properties of enhanced semiconductive plasmonic heterostructures will be discussed, where variable electromagnetic field intensities were tuned to modify electron responses, conductions, and harvesting uses.

## 4. Coupling-enhanced hybrid semiconductive-plasmonic nanomaterials

In this section, the incorporation of different nanomaterials accompanied by variable properties depending on their intrinsic atomic compositions was discussed. These nanomaterials were placed and combined with semiconductors and plasmonic materials in close physical contact. In this regard, the discussion afforded to different levels and energies associates such as: (i) molecular and chemical composition on varied substrates, (ii) polymeric shells and molecular spacers, (iii) inorganic layers within the nanoscale, (iv) multi-layered substrates in the microscale and higher sized devices toward larger scales and applications. Therefore, it variable optical approaches obtained by different technologies and methods are demonstrated. The control of the different levels permitted the interaction of chemical and physical properties that could afford new properties such as metamaterials. In this aspect, in order to discuss the generation of the new properties, improvements, and enhanced effects, the idea of coupling of quantum,^109^ semiconductive,^110^ plasmonic,^111^ and luminescence properties^112,113^ for development in energy applications was discussed. In this context, the use of the term of enhanced phenomena was related to the generation of new higher performances not expected from individual optically active components; however, at the place they were theoretically predicted in many cases by the Mie Theory of electromagnetic fields,^114^ enhanced phenomena,^115^ and by the electronic modification of electronic densities in their surroundings from molecular level to far-field distances.^116^

In addition, in order to couple the mentioned properties, the factors for tuning the sizes, shapes and geometries of nanomaterials below, within and beyond the nano-scale, should be highlighted. The control of variable emissions of halide perovskite nanocrystal arrays is based on the control of their sizes by multiplexed synthesis.^117^ In this example it was shown how the control of perovskite nanocrystals incorporated as nano-arrays resulted in improved performances within confined and reduced sizes in close contact to affect quantum properties. Therefore, it should be highlighted that smaller particles in the edge of quantum confinement regime exhibited blue-shifted emissions by the reabsorption of higher energy modes. Hence, these collective phenomena from individual interactions were used to prepare functional solar cells. Moreover, the incorporation of multi-coloured light-emitting nanocrystals in a wide range of wavelengths show potential applications for the design of optoelectronic devices and displays. In addition, the media effect and incorporation of ions and chemical species could affect the perovskites properties that should be considered in the design of the optical approach as well. Therefore, semiconductive materials accompanied by proper modified media to improve electron conductions afforded improved performances. These are helpful for the future design of materials and ideas. For example the combination of a series of organic–inorganic hybrid metal iodide perovskites with formulation AMI3, where A corresponded to methylammonium (CH_3_NH_3_^+^) or formamidinium (HC(NH_2_)_2_^+^) cations, and M was Sn or Pb permitted to tune their properties.^118^ Thus, by developing different synthetic approaches, various perovskite compositions were obtained, with the chemical formula CH_3_NH_3_Sn_1–*x*_Pb_*x*_I_3_. The nanocrystals obtained showed different properties such as optical properties, energy bandgaps, and emission intensities, controlled by the applied synthetic methodology. In this manner, different charge transports were achieved by measuring the Seebeck coefficients and Hall effects. Therefore, for Sn-doped materials, faster oxidation as p-type semiconductors and metal-like conductivity were observed.

As it could be seen in both previous examples, it was showed how it could affect the conduction and emission properties by controlling the sizes and specific atomic compositions in perovskite nanocrystals. However; these characteristics could be even modified by the incorporation of other interacting properties such as high electromagnetic fields. Under these conditions the excitations, electron ballistic, electron conduction, electronic hollow generation and related electronic processes could be affected.

In the early beginning from studies related with the spontaneous emissions of μm metallic particles at radio frequencies in presence of variable magnetic fields observed by Purcell *et al.*,^119^ to current studies and developments achieved within the nanoscale, opened new research fields such as plasmonics.^120^ In brief, plasmonic afforded to study the generation and effect of strong electromagnetic fields within the near field of variable inorganic and organic materials towards the far field, where the near field is related to the phenomena in short intervals of lengths within the nanoscale; while the Far field is related to longer lengths towards μm and even mm for higher sized substrates.^121^ In this manner, the study of coupling high electromagnetic fields generated by metallic surfaces with varied electronics properties was conducted. As for example it could be mentioned the the tuning of ultraluminescence properties from varied emitter sources such as laser fluorescent dyes.^122^ In this way, the core–shell nanoparticles are well known for enhanced luminescence properties, where each part of the nanoparticle can be tuned in order to optimize their emissions.^123,124^ These enhanced luminescence properties are based on a plasmonic effect nominated as Metal Enhanced Fluorescence (MEF). The MEF is a physical phenomenon related to the generation of a high-intensity plasmonic band and coupling with the excitation of a fluorophore that enhances the emission. This effect depends on the distance between the fluorophores and the metallic surface.^125^ The nanoarchitectures could be assembled within organized arrays in order to be placed properly within optical approaches to be evaluated for optoelectronic applications. In this regard, there are many materials under development with potential transfer towards other research fields related.^126^

Therefore, recent reported publications related to the tuning of semiconductive properties, energy losses and enhanced emissions based on the Purcell effect in photonic and plasmonic crystals should be highlighted.^127^ In this theoretical study, Surface Plasmon Polaritons (SPP) in metallic dielectric films and hollowed nanopatterned surfaces were compared. Thus, the excitonic spontaneous emission (SE) rate into a SPP cavity mode was enhanced over the vacuum decay rate through its spatially confined photon energy density (PED). Thus, an In–Au–In structure indicates that the SPP Purcell effect^128–130^ can exceed a value of 50 in the ultraviolet interval of wavelengths, contemplating energy losses. Therefore, the perspectives were highly promising for energy applications.

Moreover, in the context of analysis of energy losses, propagating signals through varied materials and coupling different energy modes, the recent reported record of the factor of Purcell determined in ultracompact hybrid plasmonic ring resonators should be highlighted.^131^ In this work, a composite hybrid plasmonic waveguide (CHPW) was developed, enabling coupled dissimilar plasmonic modes such as SPP and Total Internal Reflection (TIR) with diminished energy losses based on the incorporation of CHPW ring resonators of 2.5 μm. This approach led to the lowest propagation loss within the smaller mode area reported at the moment. Hence, this demonstration potential application for nanophotonics circuits could be contemplated.^132^ Therefore, the tuning of an antenna effect of silica nanowires on metallic surfaces should be mentioned. This approach was achieved by controlling the height of the nanowire above metallic mirrors. Thus, it was afforded to tune light scattering based on the Purcell effect from semiconductor optical antennas^133^

**Fig. 4:**
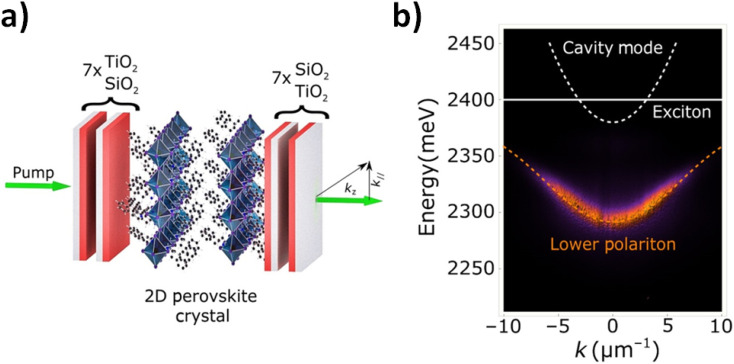
(a) Schematic of a 2D perovskite single crystal embedded in an optical cavity formed by two DBRs; in 2D perovskites, inorganic layers are separated by organic ligands realizing an effective multiple layered QW structure. (b) Energy *versus* in-plane momentum *k* photoluminescence emission from the sample represented in (a), with *k* = 2π*λ* sin *θ*, where *θ* is the emission angle; the white dashed and solid lines represent the cavity and the exciton uncoupled modes, respectively, and the orange line is a fit to the polariton lower mode with *E*_X_ = 2.395 eV, *E*_C_ = 2.385 eV, and ℏ*Ω* = 170 meV. Reprinted with permission from ref. 136 (D. Sanvitto *et al.*). Copyright 2019 Sci. Adv., Science.

Hence, these phenomena showed these types of effects, and it was extended to other research fields as nanomaterials, semiconductive materials, and hybrid materials for energy applications. For example, the excitation of plasmonic and semiconductive nanomaterial could produce other energy modes such as polaritons that could be transduced through the opto-active material. The polaritons are generated by photonics and electronic oscillation within confined resonators.^134^ By this manner, perovskites showed the generation of polaritons in lattices and necessitated studies related with optical switching of topological phases of these phenomena.^135^ In addition, it should be mentioned that these phenomena were showed non-embedded in optical cavities. This fact could be explained by its intrinsic chemical structure, geometry, and anisotropy that generated strong polaritons non-linearity, and exciton–excitons interactions energies at room temperature with potential applications for device designs. In these perspectives, as for example, it was recently reported single crystals incorporated within an optical cavity containing two Bragg reflectors (DBRs) acting as bottom/top mirrors and bare single crystals. Thus, strong polariton interactions were achieved at room temperature^136^ with an excitonic interaction constant *g*_exc_ ∼3 ± 0.5 μeV μm^2^. It should be noted that it is two orders of magnitude higher than the values of organic excitons. The promising properties for device fabrication are demonstrated (Fig. 4). In this context, it was noted that the tuning of the excited state by modifying electronic orbitals and waves associated showed implications in electronic conductions such as materials as graphene quantum dots and varied photo luminescent emitters. Thus, all types of electronic modification by induction based on the modification of the close surrounding could be a potential advance in some opto-electronic properties.^137^

The LaMer model predicts that a perovskite layer of low quality and a high defect density would result from a fast nucleation rate. Large organic cations are discovered to produce a quasi-two-dimensional (2D) perovskite rather than integrating into the lattice vacancies or the perovskite crystal structure. These cations gain from hydrophobic organic groups that result in low-dimensional perovskites, which significantly increase stability.^138^ Additionally, 2D perovskite structures with low formation energy have less ion migration and more stability. The introduction of organic cations improves band alignment and the vertical growth of the perovskite films.^139^

Before continuing the analysis of the performances and strategies related to the function of each part of the hybrid material, it should be highlighted in particular that the previous one (Fig. 4) is based on an optical resonator where varied energy modes could resonate and be amplified.^140^ So the expectation for new optically active materials is excellent. For this reason, naturally there are an increasing number of studies related about the assembling of different materials involving the control of surfaces and volumes. These apparent redundant mentions are not like that due to the high sensitivity of the phenomena associated with interphases between the different layers. As for example, the chemical modifications of semiconductors could interfere within opto-electronic responses. In addition, it should be noted that it is not required for higher sized molecular structures to affect them; it is just needed to involve small molecules and atoms with particular properties such as high electronic densities and polarizability.^141^ In this regard, by improving surface cleaning with strong and pure acids, and adding multilayered targeted silanes, nitrides coupling agents for passivation between other chemical modifications, it was recently reported efficient heterojunction back contact solar cells with a certified efficiency of 27.09% using a laser patterning technique.^142^ From this research, it was found that recombination losses primarily arise from the hole-selective contact region and polarity boundaries. It was demonstrated wafer edge becomes the main channel for current density loss caused by carrier recombination once electrical shading around the electron-selective contact region is mitigated. This level of control was achieved by multilayered incorporations involving multi-step wet chemical cleaning, chemical vapor deposition (CVD), laser ablation, PVD, and metallization processes. Therefore, the required advanced nanocrystalline passivating contacts and wafer edge passivation accompanied by meticulous optimization of front anti-reflection coating and rear reflectors were achieved. By this manner, it was chellenged similar efficiencies reported by other researchers at the same time.

Moreover, and focusing on the previous examples, the intrinsic properties of perovskites could be highly sensitive to their close surrounding. Thus, there was strong excitation between plasmons and excitons within colloidal halide perovskite nanocrystals near a metal film.^143^ This coupling regime based on mixed plasmon-exciton formation was nominated as plexcitons; and generated from colloidal perovskite nanocrystals in close proximity to chemically functionalized metallic surfaces. Therefore, strong oscillator strengths from quantum confined properties of perovskites were coupled with high-energy electronic oscillations of metallic surfaces. Both different and high-energy modes afforded enhanced properties without the need for both the types of materials to be in direct contact. Finally, it should be highlighted that the Rabi splitting energies varied depending on shapes and dimensions. These plasmonic–perovskite structures were considered as potential materials for studying light-matter interactions at the nanoscale towards the micro- and macro-scale. In this manner, these strong interactions allowed the design and fabrication of enhanced perovskite metallic photodetectors based on plasmonics.^144^ Enhanced properties accompanied with improved performances were achieved by combining gold tringles nanoantennas with CH_3_NH_2_PbI_3_ film. The experimental results were correlated as well with theoretical calculations that enabled understanding and explaining the mechanism of the proposed coupled energy modes. In a similar manner, the combination of both silver island films (SIF) and CH_3_NH_3_PbI_3_ perovskite films obtained on the surface of SIF has been recently studied. The surface morphology showed an influence on the optical effect of the substrate, as predicted previously. A redshift in the absorption spectrum and increased intensity of emission accompanied by fluorescence lifetime decay shortening were observed. This result showed potential uses to enhance the intensity of light emitting diodes; and the lifetime of charge carriers within coupled perovskites for photovoltaics applications such as solar cells was improved.^145^

It could be highlighted that plasmonic–perovskite in combination as well with other nanomaterials afforded to be considered in designs for solar energy applications such as for photovoltaic devices. Thus, improved performances were explained by the generation of hot electron in plasmonic nanostructures. As for examples, based on a tuned profile composed by ITO/TiO_2_ NPs/perovskite/Au NPs was produced a high energy charge potential profile across the cross section of perovskites solar cell before and after the plasmonic layer recorded by imaging of irradiated energy fields.^146^ Therefore, the mechanism of electron dynamics and energy losses within solar cells can be explained and understood. A new paradigm for future advances in solar cell fabrication contemplating passivation and naturally further nanochemistry and nanoplasmonics was opened. In the bottom up, it was highlighted these mentioned variables such as highlighted research mentioning dual-interface passivation to improve the efficiency and stability of inverted flexible perovskite solar cells by *in situ* constructing 2D/3D/2D perovskite double heterojunctions.^147^ By the incorporation of benzenebutanammonium iodide (PBAI) as small spacer molecule, it was made 2D/3D/2D perovskite double heterojunctions, affording to 2D and 3D perovskites bottom ups. As a result, inverted double heterojunctions exhibited a high power conversion efficiency (PCE) of 24.08% with a low *V*_oc_ loss of 0.37 V due to the minimal nonradiative recombination by dual-interface passivation. Thus, it was noted recently main variables considered to achieve very good performances in comparison to contemporaneous investigations.

In this section it was leaded to propose new emergent approaches by the incorporation of high energy electromagnetic fields within the near field towards far field applications such as enhanced photoluminescence and energy solar cells applications, highlighting the importance of the different components, surfaces and optically active materials joined.^148^ It was introduced to the discussion about how it could be affected the electronics, quantum properties, energy modes and charge transportation through coupling plasmonic substrates with other types of nanomaterials such as varied nature of semiconductors, perovskites nanoparticles, and hybrid organic/inorganic materials too. Thus, in the next section, current challenges within these multidisciplinary research fields will be discussed.

## 5. Solar cell applications

In the precedent sections developed were discussed different variables that influenced the efficiency of tuned materials for energy applications. However, it was shown many research related with advances for the study and application within the next technology approach. In this regard, it is mentioned many insights such as from ultraluminescent materials to applied optics, nano-bio-optics, optoelectronics and more. In this context, solar cells and applied energy uses are of high interest with social impact. Therefore, it was afforded to opto-stimulations, conductions, harvesting, and storage of varied energy modes. So, the nanophotonics factor in common was the efficiency of the transduced signal such as photons or electrons. Thus, the energy losses and stability of the material constituents were involved in all the mentioned phenomena that affected even without contemplating their participation. In this manner, looking for improved efficiencies in solar cells by designing new materials should be contemplated, for example, intrinsic fundamental limits of nanophotonic light trapping.^149^ As it is known in the bulk the standard theory of light trapping demonstrated that absorption enhancement in a medium cannot exceed a factor of 4*n*^2^/sin^2^ *θ*. Thus, *n* is the refractive index of the active layer, and *θ* is the angle of emission cone in the medium cell surrounding.^150^ However, this model did not explain the nanophotonic behaviours. For these reasons, it was proposed a new theory contemplating a statistical temporal coupled mode theory of light trapping by electromagnetic physics considerations; where it was showed that it could be overcome the limits by the generation of optical modes of deep subwavelength scale field confinement. In brief, according to this theory, the incorporation of embedded low index absorptive nanomaterials can drastically enhance light absorption beyond the conventional limits in bulk. This theory was extended to nano-patterned plasmonic structures^151^ Thus, there actually exists a new research field focused on nanophotonics and photovoltaics.

So, at each step of the light conversion was optimized for real improvements in the targeted applications based on individual enhanced phenomena from the molecular level to nano-, and micro-scales. This is a concept that was repeated along this communication that it could be found as factor in common in all research developed in the last years. In addition, the incorporation of perovskites within plasmonic solar cells opened as well to new strategies of study and challenges. Based on experimental and theoretical studies, the plasmonic approaches to modify the energy band gaps of semiconductors in the active layer should be mentioned, and it diminished the binding excitation energy affording a higher power conversion efficiency (PCE).^152^ By this manner, the addition of plasmonic nanomaterials within perovskite solar cells (PSCs) has increased their efficiencies based on surface plasmon resonances. The hot electrons produced by plasmonic nanoparticle resonances can be directly injected into the perovskite environment to improve the mobility of photo-generated electrons by filling the trap states.^153,154^ This made a huge difference in the electronic generation, conductions, and transmission. It is a high energy electromagnetic field that modifies the electronic matter constitution. Thus, sizes, shapes, and metallic nanostructures applied could lead to tunable surface chemistries and resonance wavelengths. Moreover, these tunable optical properties could be coupled with other optical properties as it was discussed in the previous section for further studies. For this reason, this research field is currently of high impact with direct implication with the fabrication of the next generation of solar cells.^155^ In this way, the spin-coating technique is the most well-known technique used to deposit multiple perovskite solar cells (PSCs) and nanoparticles in order to evaluate high efficiencies for larger scale viability. In a similar manner, a broad range of other manufacturing techniques such as drop casting, spray coating, ultrasonic spray coating, slot die coating, electrodeposition, CVD, thermal vapor deposition, vacuum deposition, screen printing, and ink-jet printing^156–159^ were used, and could be taken into account for further studies.

These phenomena show influence as well in other variables previously mentioned such stability of modified halide perovskite surfaces.^160^ Thus, it was avoided the contact between perovskite defects and oxygen by salts formation; however similar approaches could be proposed combining plasmonic nanomaterials. Moreover, as it is known that luminescence effects could be tuned through InGaP/GaAs by current voltage analysis within tandem solar cells.^161^ Thus, the incorporation of plasmonics engineering such as Metal Enhanced Fluorescence (MEF)^162,163^ could affect the different mechanism related to the efficiency of solar cells. In this context, it is noted the incorporation of quantum dots emitters combined with other semiconductors materials such TiO_2_ nanoparticles with particular emission properties as light sensitizers for solar cells applications.^164^ As well, it should be mentioned that previously it was reported enhancements of single quantum dots. It could be mentioned not only fluorophores,^165^ but also CdSe/ZnS quantum dots or CdSe nanorods by MEF^166,167^

In this related field where the light absorption and up on conversion is of high interest, it was highlighted that the enhancement factors are affected by further variables such as wavelenghts and energy involved from the basal to the excited state accompanied to the generation of different opto-electronic active compounds. Thus, it could be noted the wavelength-dependent metal-enhanced fluorescence biosensors *via* resonance energy transfer modulation could affect further electronic conductions.^168^

In addition, combining nanoparticles with other optically active nanomaterials such as perovskites showed improved properties and performances for solar cell design and fabrication. Thus, it was showed that it is possible to manage photons and charge carriers through the hybrid nanomaterial; where each component had a defined function.

In recent years, researchers have focused their attention on ‘multiple exciton generation’ and ‘hot carriers in quantum and enhanced luminescent confined nanostructures. These studies are expected to provide a solution to more efficient photovoltaic devices required for the future. In nanostructures, low defect densities lead to reduced recombinations, and in turn, increased open circuit voltages and short-circuit currents. These facts are part of the expected future improvements.^169^

**Fig. 5:**
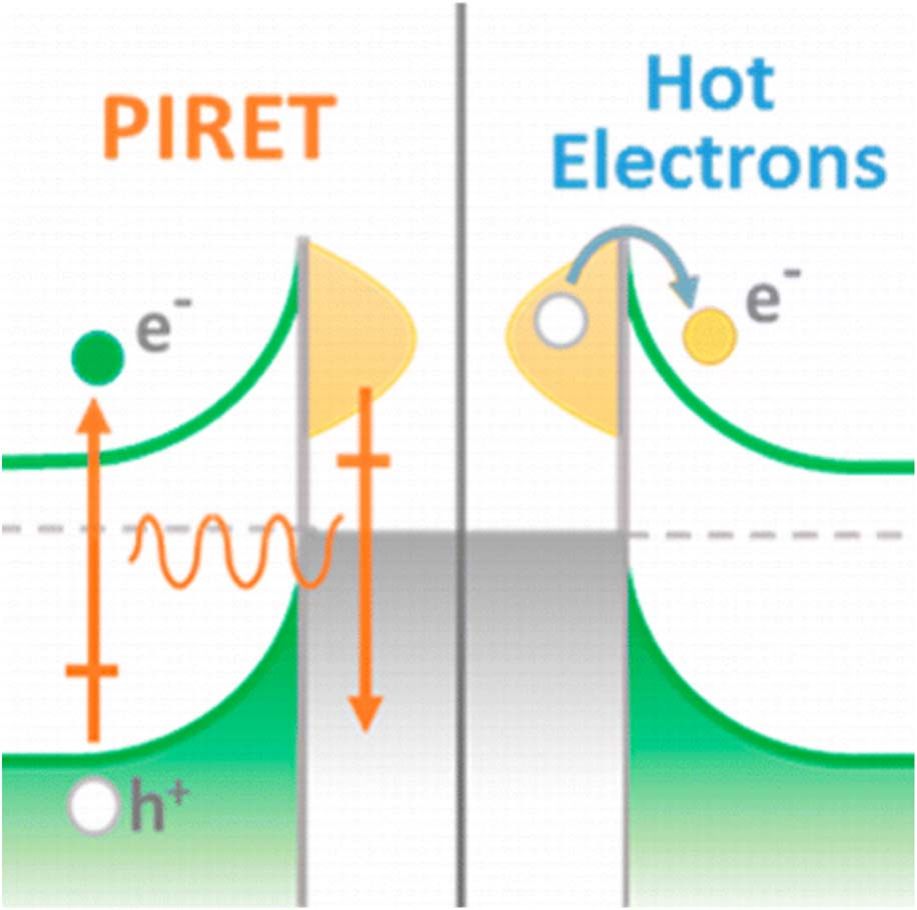
Schema of the electronic conductions by plasmonic metals *via* charge carrier excitation in semiconductors through plasmon-induced resonance energy transfer (PIRET) and hot electron injection processes. Reprinted with permission from ref. 176 (N. Wu *et al.*). Copyright 2015 J. Phys. Chem. C, ACS.

The surface plasmons can be tuned by controlling the shape, size, and dielectric environment of the nanomaterials. It is known that the plasmonic intensity is related to the sizes and shapes of nanoparticles, and for a given nanoparticle shape, higher intensities are generated from higher sized nanoparticles.^170^ CaTiO_3_ perovskites could generate electron holes and charge carriers by light excitation. Then, the carrier extraction occurred through an interaction with electron/hole transport layer, while carrier injections occurred at the interfaces of electrode layers and charge transport layers. Therefore, the performance in these phenomena could affect drastically the efficiency. Thus, the accurate addition of plasmonic nanomaterials could affect the electron transport through the material. This effect was explained by their high energy electromagnetic fields interactions with perovskites and charge transport layer that could produce higher electron charge carriers and transport in comparison to thermal deactivation as well as by other non-conductive electron losses. In this context, it was described as a phenomenon generated by augmented absorption cross sections of opto-active particles and hot electron pair generation faster than in the absence of plasmonic nanoparticles.^171^ In this manner, energy losses were drastically diminished and increased light harvesting was obtained. It should be mentioned the implication of not only the sum of single plasmonics events; as well it should be contemplated collective oscillations related with Enhanced Plasmonics (EP) in similar manner as it was observed for luminescence emissions.^172–174^ The EP is actually incorporated in many optically active substrates as the waveguide of light and electrons affording real improvements. However, for real-time solar cell applications as well as other related energy material optimization, further research is ongoing.^175^ In this context, it was proposed unless four mechanisms that could participate in the enhanced coupled plasmonics efficiencies within semi-conductors heterojunctions as it is stated: (i) plasmonics Hot Electrons Transfers (HET) from metal to semiconductors; (ii) Direct Electron Transfer (DET) nominated as well as Hot Electron Injections (HEI); (iii) Plasmon Induced Charge Transfer (PICT) transition across the interface; and (iv) Photonic Enhancement (PE) of Resonance Energy Transfer (RET) (Fig. 5).^176^

In this perspective, there are a large number of possibilities and combinations of various plasmonic nanomaterials joined to perovskites within different optical approaches. It is known that hot electrons and excitons could be generated by plasmonic coupling with other optically active materials.^177^ By this manner, it could be tuned as well different properties as well as targeted applications. For example, plasmonic nanoparticles were assembled with perovskites, as light-harvesting enhancers within solar cells.^178^ This study was developed theoretically, and it afforded to predict the magnitude of the absorption enhancement embedding metallic nanoparticles with perovskites. Thus, higher efficiencies were obtained from thinner films than those normally used. Hence, the collection of photo-carriers from the confined device studied was facilitated. Potential toxic materials can be reduced in future cells. However, it should be highlighted that the approach applied was embedding both components together; and other types of strategies could be summed for future studies and evaluation of efficiencies of new material designs. So, many variables could be taken into account related to set-ups and spatial orientation of the components.

Therefore, core–shell refractory plasmonic nanoparticles have been recently reported. These nanoarchitectures acted as excellent nanoantennas in order to increase the efficiency of lead-free perovskite solar cells (PSCs).^179^ In this approach, SiO_2_ was used to form a shell due to its high refractive index and low extinction coefficient. ZrN/SiO_2_ nanoarchitectures were modified with PSCs. Thus, the decoration with ZrN nanoparticles boosts the power conversion efficiency (PCE) of the PSCs from 12.9% to 17.0%; while the use of the ZrN/SiO_2_ core–shell generated an amplified enhancement to 20.0%. Thus, it was demonstrated how the accurate control within the nanoscale, below and beyond, it could affect drastically the effect on the far field of the plasmonic material such as by MEF-FRET pathways.^180^

**Fig. 6:**
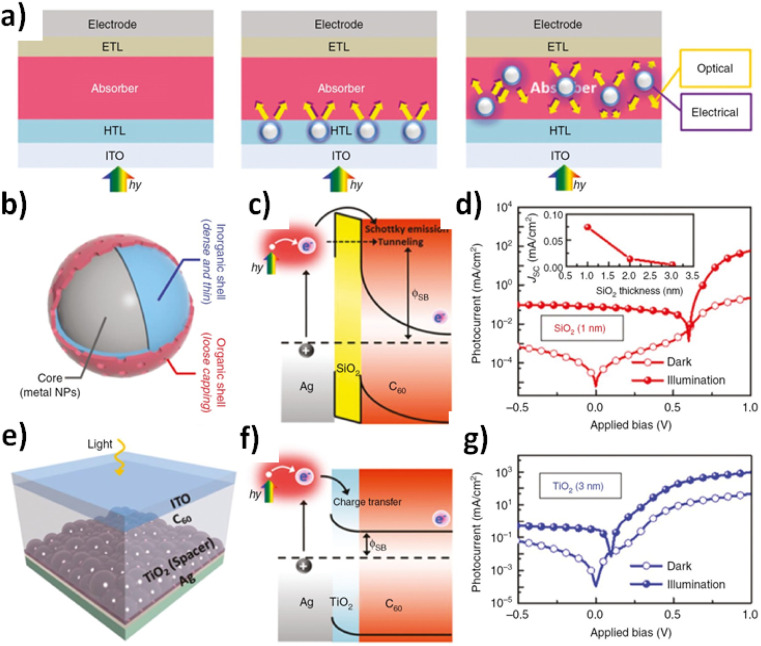
Multiple synergistic effects of optical absorption enhancement and electrical characteristics occurred in organic solar cells (OSCs) with the incorporation of plasmonic metal NPs: (a) schematic of the plasmonic solar cell architectures with different spatial locations of metal nanostructures. (b) Core–bishell design concept for metal nanostructures embedded within an active layer. (c) Nanoplasmonic device structure consisting of Ag/TiO_2_/C_60_ and Ag/SiO_2_/C_60_ stacked films to simulate the core–bishell designs of Ag@TiO_2_@Pa and Ag@SiO_2_@Pa, respectively. (d) Energy diagram of electron excitation based on between-band transfer for the SiO_2_ interface. (e) Energy band diagram of metal–insulator–semiconductor for the device with a TiO_2_ layer. Current–voltage characteristics in the dark and under simulated AM 1.5G solar illumination of a typical device with 1 nm of SiO_2_ spacer shell (f) and 3 nm of TiO_2_ deposition (g) between nanostructured Ag film and C_60_. The inset of (d) demonstrated the dependence of SiO_2_ thickness on short-circuit current. Reprinted with permission from ref. 183 (H.-Bo Sun *et al.*). Copyright 2020 Nanophotonics, De Grutyer.

It should be mentioned the high sensitivity of Metal Enhanced Fluorescence (MEF) within shorter silica spacers as it was previously reported from accurate control of variable lengths deposed on silver^181^ and gold nanoparticles.^182^ These values were in the range of 5–15 nm length for both core–shell nanomaterials; however, different profiles of plasmonic intensities and MEF factors were determined depending on the core template used. Higher values were recorded from silver than gold; however, for biocompatible application, gold was chosen due to its low toxicity under controlled conditions. Moreover, it should be mentioned that within all these types of tuneable nanomaterials, the hybrid nanocomposites showed to be the best option to exploit improved efficiencies from the molecular level towards the nanoscale and beyond. Therefore, from the bulk it was possible to tune lower energy energy losses and higher efficiencies from the bulk. In this regard, the plasmon-enhanced approaches of organic and perovskite solar cells by incorporating metal nanoparticles (Fig. 6) should be highlighted.^183^

It is highlighted the improved performances non-recorded before by adding new core–shell nanoarchitectures.^184^ These types of structures showed improved performances due to the capability to tune the optical properties with an accurate control of lengths and multi-layered additions related to different coupled photo-physical properties.^185^ In this regard, there is an open window of potential further fundamental and applied research considering coupled phenomena as well. It is mentioned recent studies related with other organic material with confined electronic properties with potential interactions with the near field from metallic surfaces and nanoparticles such as carbon quantum dots. These types of quantum nanomaterials showed high quantum yields^186^ with potential applications for bioimaging^187^ and bioconjugation applications.^188^ Further studies are of interest focusing on biocompatibility,^189^ low-cost procedures in the fabrication, and incorporation in nanomaterials with high social impact such as solar cells.^190^

Moreover, from different studies and approaches developed, it was inquiered about what is the most versatile and low cost fabrication method to apply. So, another extra factor such manufacturing processes for solar cells could be contemplated depending of the state of the art of the technology to scale up modified substrates and devices. Moreover, the importance of the plasmonic nanomaterials within the optically active set up where it could be incorporated should be mentioned. Thus, perovskite solar cells incorporated varied nano-plasmonic materials mainly in the active,^191^ and buffered layers.^192^ Therefore, from the active layers were explained improved results based on Enhanced Charge Generation (ECG),^193^ local surface plasmon resonance LSPR,^194^ and Enhanced Charge Transfer (ECT)^195^ While, by incorporation of the nanoparticles in the buffer layer, it was developed diminished resistance,^196^ and photon recycling scattering.^197^

In addition it could incorporated further mechanisms below the nanoscale that could be tuned for improved and targeted mechanism pathways such as plasmonics coupling within core–bi-shell structure.^198^ This approach it was formed by a silver core (Ag) covered with titania (TiO_2_ NPs) and an extra organic layer of benzoic acid–fullerene (Ag@TiO_2_@Pa). The optical set-up showed improved properties based on synergistic effects (optical and electrical phenomena). For example, organic solar cells led to an enhancement of 12–20% with a maximal power conversion efficiency of 13%, while the plasmonic core–bi-shell nanostructure produced an enhancement by 10% from 18.4% to 20.2%. The constitution of this bi-layered core–shell permitted an improved short circuit current from within the nanoparticle and through the cell material by light excitation with electronic current generation.

Thus, core–shell nanoarchitectures showed the capability to modify solid substrates that affected technologies where multilayered approaches such as heterojunctions,^199^ Light Emitting Devices (LEDs),^200^ and solar cells^201^ are used. From other scale and point of view, as well the core–shells act as photovoltaic nanometre-scale cells embedded in photo-cross linkable organic semiconductors.^202^ These recent insights are still being under study towards new developments. The incorporation of these nanoarchitectures augmented the total surface of contact and it improves the exchange of energy in their inter-phases. Thus, it was improved phenomena where electron densities are involved resulting in higher performances in comparison to non-modified substrates. Thus, it is highlighted an improved large-scale integration due to the capability to manufacture these Nanomaterials by different techniques and methods.^203^

In this manner, the importance of the targeted function of each component of the confined nano-circuit showed improved performances. Thus, other possible combinations of hybrid organic–inorganic nanomaterials were reported, where each material contributed with their intrinsic properties that in combination could show synergistic effects, coupled phenomena, and enhanced effects. It is highlighted a recent report related with hybrid plasmonic material for solar cells using a photonic–plasmonic strategy based on the improved light absorption in the active layer.^204^ This approach was formed by a modified conventional planar plasmonic solar cell (PSC) with a nano-patterned substrate with 100 nm semi-elliptical gold geometries joined to other components such as; Au/CuSCN/perovskite/TiO_2_/ITO/PDMS/air. In this manner the absorption of light at varied wavelengths and electromagnetic fields generated was evaluated. In this way, it was produced a high absorption accompanied with high electromagnetic fields within the active layer that afforded to trap light inside perovskites. Consequently, a high current density was produced in the cell accompanied by a drastic reduction in energy losses. It was achieved an increase of the short-circuit current density (*J*_sc_) from 18.63 mA cm^−2^ for the planar structure without nano-patterning modification to 23.5 mA cm^−2^ for the nano-patterned substrate. So, the increased *J*_sc_ and open-circuit voltage (*V*_oc_) caused by and augmentation of the carrier collection, generated a Power Conversion Efficiency (PCE) increases from 14.62 to 19.54% respectively.

As it was discussed previously, opto-electronics properties of solar cell materials by the incorporation of plasmonics structures as opto-active substrates and perovskites as excellent electro-active materials could be tuned and improved. It was achieved the different variables from the intrinsic nanomaterials applied accurately assembled to varied configurations and set-ups of final substrates obtained. However, there are other organic and inorganic materials that are currently in constant developments as well.

**Fig. 7:**
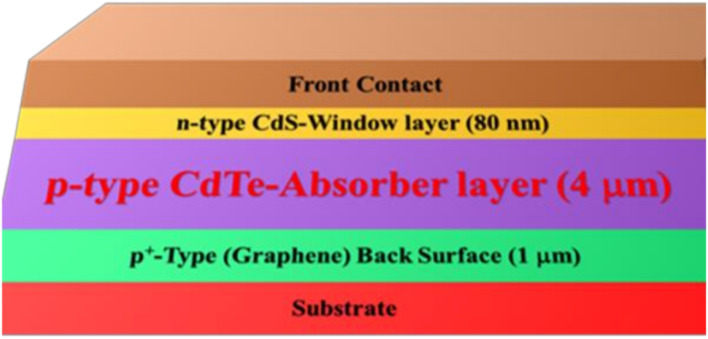
Device structure of the graphene back surface-based CdTe solar cell. Reprinted with permission from ref. 207 (B. Pant *et al.*). Copyright 2021 Molecules, MDPI.

Finally, other semiconductive nanomaterials applied for solar cells should be mentioned. These materials allowed electron conductions, electron shuttling, and generation of new modes of energies such as plasmonics, and afforded to coupling different properties for improved efficiencies. It could be mentioned studies by the incorporation of graphene quantum dots incorporated within conducting polymer/porous Si hybrid solar cells with titanium oxide passivation layer with higher stabilities and performances.^205^ Graphene, a high opto-electro-active organic material, with similar conductive properties of Copper and enhanced properties joining both materials within composites,^206^ was applied as a back surface material in solar cells. This phenomenon is related to the diminution of electron recombination and energy loss from the back layer of the substrate. It was reported theoretical investigations of graphene as a back surface field layer on the performance of cadmium telluride solar cell (Fig. 7).^207^ The results showed very good performances with the highest short-circuit current (ISC) of 2.09 A, a power conversion efficiency of 15%, and a quantum efficiency (QE) of 85% with a carrier Lifetime of 1 × 10^3^ μs.

Herein, the importance of theoretical calculations that afforded to useful conclusions to design materials for solar cells should be highlighted. In this aspect, highly conjugated organic molecules were applied; small molecular organic energy acceptors for organic solar cell fabrication were also studied and used. Therefore, performances of solar cells were calculated by the evaluation of varied chemical structures.^208^ However, it should be mentioned that highly conjugated carbon chemical structures as carbone allotropes, different from graphene, were used in interesting approaches for optical activity due to their particular properties as well. This is the case of fullerenes with high electronic densities generated from spherical carbon-based chemical structures within the nanoscale.^209^ Therefore, it has been recently reported that non-fullerenes acceptors with reduced non-radiative voltage losses (Δ*V*_nr_) are explained by a different mechanism in comparison to fullerenes. Different mechanism of non-radiative voltage losses in organic solar cells were described.^210^ Thus, it was discussed in contrast to the energy gap law that using non-fullerenes acceptors, a correlation with the energies of charge-transfer electronic states at donor–acceptor interfaces was not shown as it was described for fullerenes. This explanation was based on the role of the thermal population of local exciton states in low-Δ*V*_nr_ systems. The pristine no-fullerene material determined the photoluminescence yield of the lower limit of Δ*V*_nr_; so it was diminished charge generation losses. A new design of the incorporation of donor:acceptor materials with improved Quantum Yields (QY) and overlapped optical absorption/emission bands until the Near-Infrared Region (NIR) was proposed. Moreover, the addition of varied opto-electrical active materials could be tuned each part of the multilayered materials such as electron shuttle, metallic conductive materials, and organic semiconductive materials; from where there is a generation of new optically active functional materials.^211^

From the different research works discussed, many mechanisms and strategies used to develop solar cells depending on the nanomaterials, and accurate positioning of them in the support substrate were observed. The important factor related to solar cell scale-up and manufacturing was mentioned. Thus, the review and analysis of variables from the nano-, and micro-scale to bulk phenomena towards high efficiency of solar cells such as using perovskites were showed to be all important.^212^ Thus, it was accompanied by the evaluation of parameters related to the efficiency and stability of solar cells.

Moreover, other new variables incorporated such as alternative green solutions or trends for the fabrication of these types of materials should be mentioned. Research studies such as the fabrication of green efficient perovskite solar cells from recycled car batteries,^213^ and incorporation of biocompatible nanomaterials such as modified chitosan-based gel polymer electrolytes incorporated with potassium iodide for dye-sensitized solar cell assembly were mentioned.^214^ These approaches are related with challenges from Green Chemistry^215^ developments which are currently in progress; however these themes exceeded the extension of the present communication. But, it could be taken into account for future discussions and developments. In this regard, and opening concluding remarks maybe it should be highlighted biocompatible and wearables uses that require higher sensitivities and solar light, electric conductions and opto-electronic based phenomena require further Research. In this context, it should be noted the interest in the previous discussion towards physical mechanism studies in relation to of surface plasmons, FRET, and plasmon-enhanced FRET coupling. From there, different nano-systems and set ups are still being under consideration, such as core–shell structures, nanorod, ultrathin films, waveguides, optical fibers, chips, devices, and heterojunctions. The coupling of optical biostructures as well as consideration of green synthetic materials with perspectives to biocompatible materials and related properties has potential applications in optoelectronic devices, solar cells, biodetectors, sensors, *etc.*^216^

## 6. Conclusions and future perspectives

The perspectives of semiconductor materials are broadly expanded at different levels, from the quantum and nanoscale towards nanodevices and microdevices and beyond for photonics and solar energy applications. With this perspective, many developments of heterojunctions and hybrid nanomaterials that led to improved properties were achieved. Thus, varied semiconductive materials were used for these studies; however, it was not all contemplated with the best known nanomaterials used. In this perspective, the main challenge for photonic applications, and in particular for solar application, is always the efficiency and stability of the material developed. For the near future it could be proposed other approaches by varying: (i) bottom up, (ii) varied properties of materials; (iii) multi-layered incorporations; (iv) enhanced resonant electromagnetic fields of varied material constitution, such as enhanced plasmonics; (v) coupling of electronic, magnetic and quantum properties for improved and enhanced absorption, storage and emission.^217,218^ These are potential phenomena involved within photonics and photovoltaics processes that could be of high impact on expected performances.

The analysis of enhanced plasmonics pathways controlling the nanoscale and related effects on the performance of perovskite solar cells showed a clear direction through surface recombination and short-circuiting designs. Thus different behaviors could be considered from recent studies, highlighting key improvements to consider in future approaches as well.^219^ The stability of absorption was further optimized, achieving a remarkable 15% absorption enhancement compared to the reference TiO_2_–MAPbI_3_–spiro cell. This was not a neglected improvement to highlight. In this context, it should be mentioned that in all the developments previously discussed, classical and non-classical light signaling with improved efficiencies was showed, from the nanoscale and below towards the micro-, and macroscale. The modification of the optical set up showed good improvements. It was demonstrated that uniform nano-plasmonics arrays achieved wide-band absorption across the visible and near-infrared regions, reaching up to 100% of the visible range spectrum compared to 92% for single mode of a nanostructured disk. Therefore, low signaling from bulk in some cases was attributed to energy losses explained from molecular and nano-surfaces with topographical irregularities. Enhancements and coupling of properties are dependent on short lengths within the nanoscale and quantum dimension. Thus, varied phenomena that would be evaluated with potential impact on these research fields such as FRET,^220^ MEF, FRET–MEF coupling, EP, and EP coupling with other properties could be highlighted.^221^ However, it should be noted that these mentioned properties could be contemplated for future developments, as well as other ones should be added. Therefore, in the next generation of semiconductive materials, heterojunctions, and new bottom ups, the control of properties within the quantum and nanoscale could be the solution for many queries and challenges as well. Therefore, there are many variables and combinations that are still missing to be developed. Hence, new possibilities and new ongoing research works are in progress as well as other ones should be proposed.

All these mentioned variables could be developed by different techniques such as wet chemistry methods and lithography techniques. From the wet chemistry point view, the control of the molecular incorporation is directly related to the chemistry of colloids; while the control of the nanoscale could be afforded by the lithography as well as colloidal dispersions. The state of the art of other techniques, such as vapor deposition, sputtering, and controlled varied nanomaterial deposition should be added. Therefore, these multidisciplinary research fields and industries will allow the next generation of solar energy materials. In addition, the material science, with design and synthesis of new materials and metamaterials, should be contemplated. Therefore, all these future developments could even be improved with resonant approaches where the signal involved is amplified as a Laser based optical system. By this manner, other new ideas and proofs of concepts could be joined as well. Within all these topics, the green chemistry point of view, the upscale of hetero-structures, and manufacturing as well as cost issues will still be in discussion.

These different factors and variables that could be involved in the near future come from the analysis of recent publications. In this large variety of possibilities, the improvements in the near future based on new nanomaterials for energy storage were highlighted These nanomaterials are based on the high surface volume ratio and associated with high energy density and enhanced opto-responsive sensitivity properties. This application is very important from the practical point of view after energy collection; and for their transference in new technology such as wearables, smart responsive surfaces, and storage devices at different levels.^222^

The use of silicon solar cells for improved properties by studies related to the optical properties of heterostructures is still of interest due to the excellent characteristics of this material that joined other ones with different bulk properties. Thus, for example, a study about the refractive index on coupling structures for silicon solar cells has been recently reported.^223^ In this article, it was demonstrated theoretically that the refractive index of the light trapping structure strongly influenced the light trapping behavior. Moreover, the use of other types of polymers coupled with well-known and efficient semiconductors such as perovskites was reported. The incorporation of star-shaped polymers within inverted perovskite solar cells with very high Efficiency and stability has been demonstrated recently.^224^ Graphene, a well-incorporated organic semiconductor on the market, is actually used for new plasmonic heterojunction approaches and emerging photovoltaics could be involved too.^225^ The electron coupling from the nanoscale to the resultant interfacial energy band reorganization created enhanced performances by light excitation. This result was obtained by controlling the 2D and 3D structures of the materials incorporated. Thus, as it has been discussed previously, the optical set up and accurate positioning of all the components are essential variables to control for the targeted tuning of properties. In this perspective, the holographic low concentration optical system increasing light collection efficiency of regular solar panels has been recently published as well.^226^ In this work, an holographic light collector at the place of conventional modules with silicon or gallium arsenide photovoltaic cells was proposed, which produced energy yields of 4.5%, 4.1%; and 3.8% with PV panels deployed with two-axis, and single-axis tracking systems; and without tracking systems, respectively. Topics such as stability, green chemistry methods and techniques used, and efficiencies are still in discussion as well. Therefore, efficient and environment-friendly perovskite solar cells *via* embedding plasmonic nanoparticles on realistic device architectures were developed.^227^

The improved efficiencies and properties of plasmonic nanoparticles afforded the incorporation of new bimetallic nanoarchitectures coupled to other semiconductive nanomaterials such as TiO_2_,^228^ and resonant plasmonic Heterostructures for optically thin silicon solar cells approaches^229^ Therefore, well-established substrates such as silicon solar cells for these studies are being modified as well as new designs, bottom ups and optical set-up are considered combining all the best semiconductive materials.^230^ In addition, new materials are being developed and proposed, for example metamaterials. In this perspective, the results related to nearly perfect absorbers made from a natural hyperbolic material for harvesting solar energy have been recently reported^231^ This is just a mention of some of the materials that could be involved; however, it is important to show the diversity of materials and designs focused always on the same main factors involved that affect their efficiencies. In many cases, there are factors in common that could be tuned by different manners due to their different intrinsic properties. Such is the case of quantum nanomaterials for quantum photonics applications and beyond.^232^

In order to expand applications and develop new technologies, it was afforded to other innovative research and current uses; such light generation by plasmonics light-emitting diodes (P-LEDs) based on two-dimensional lead-free perovskites,^233^ and perovskite-coated window glasses as semi-transparent luminescent solar concentrators.^234^ In addition, to highlight the importance and impact of radiation matter interactions it could be mentioned the developments of special photonics materials for solar sails through space,^235^ highlighting low weight materials,^236^ and incorporation of the best semiconductive properties such as perovskite solar cells for space uses.^237^ Moreover, further developments highlighting the control of the Nanoscale as multi-layered core–shell nanoparticles coupled to perovskites have recently showed new mechanism and light pathways promising even better energy applications. Thus, enhancing the photovoltaic efficiency in half-tandem MAPbI_3_/MASnI_3_ perovskite solar cells with triple core–shell plasmonic nanoparticles was highlighted.^238^ The importance of all the themes and topics opened for further discussion towards new designs incorporating the tuning of advanced nano-optics was presented.^239^

## Data availability

The information and data that support the development of this review manuscript could be provided by correspondent author A. G. B. up on request from third parties respecting applied restrictions, licenses, and data under current study.

## Conflicts of interest

There are no conflicts to declare.
